# Roles of Bronchopulmonary C-fibers in airway Hyperresponsiveness and airway remodeling induced by house dust mite

**DOI:** 10.1186/s12931-017-0677-8

**Published:** 2017-11-29

**Authors:** Zhimei Yang, Jianguo Zhuang, Lei Zhao, Xiuping Gao, Zhengxiu Luo, Enmei Liu, Fadi Xu, Zhou Fu

**Affiliations:** 1 Pediatrics Research Institute, Ministry of Education Key Laboratory of Child Development and Disorders, Children’s Hospital of Chongqing Medical University, No.136, Zhong Shan 2nd Road, Yuzhong District, Chongqing, 400014 China; 2 Department of Respiratory Medicine, Children’s Hospital of Chongqing Medical University, Chongqing, China; 3 Pathophysiology Program, Lovelace Respiratory Research Institute, Albuquerque, NM USA

**Keywords:** Substance P, Airway smooth muscle, Airway inflammation, Airway epithelial cells

## Abstract

**Background:**

Asthma is characterized by chronic airway inflammation, airway hyperresponsiveness (AHR), and airway remodeling. While exposure of house dust mites (HDM) is a common cause of asthma, the pathogenesis of the HDM-induced asthma is not fully understood. Bronchopulmonary C-fibers (PCFs) contribute to the neurogenic inflammation, viral infection induced-persistent AHR, and ovalbumin induced collagen deposition largely via releasing neuropeptides, such as substance P (SP). However, PCF roles in the pathogenesis of the HDM-induced asthma remain unexplored. The goal of this study was to determine what role PCFs played in generating these characteristics.

**Methods:**

We compared the following variables among the PCF-intact and -degenerated BALB/c mice with and without chronic HDM exposure (four groups): 1) AHR and pulmonary SP; 2) airway smooth muscle (ASM) mass; 3) pulmonary inflammatory cells; and 4) epithelium thickening and mucus secretion.

**Results:**

We found that HDM evoked AHR associated with upregulation of pulmonary SP and inflammation, ASM mass increase, epithelium thickenings, and mucus hypersecretion. PCF degeneration decreased the HDM-induced changes in AHR, pulmonary SP and inflammation, and ASM mass, but failed to significantly affect the epithelium thickening and mucus hypersecretion.

**Conclusion:**

Our data suggest an involvement of PCFs in the mechanisms by which HDM induces allergic asthma via airway inflammation, AHR, and airway remodeling.

## Background

Asthma is an airway chronic inflammatory disease that is mainly characterized by airway inflammation, airway hyperresponsiveness (AHR), and airway remodeling [1–3]. AHR could result from excessive contraction of airway smooth muscle (ASM), thickening of the airway wall, and over-sensitization of sensory nerves [4], while airway remodeling stems from an enhanced ASM mass and thickened abnormal epithelium with mucus gland hypertrophy [5, 6]. Most asthma begins in childhood with sensitized airway responses to common aeroallergens, such as house dust mite (HDM), cockroaches, animal dander, fungi and pollens [2, 7]. Animal model of the HDM-induced asthma has been well established with the key features including AHR, airway remodeling (ASM thickening and mucus hypersecretion), and airway inflammation (abnormal airway eosinophilia) [8, 9]. However, it is unclear how HDM affects epithelial cells, such as Clara cells and ciliated cells, and how HDM induces the airway inflammation, AHR, and airway remodeling.

Bronchopulmonary C-fibers (PCFs) represent ∼80% of the vagal bronchopulmonary afferents innervating the airways and lungs. PCFs have been reported to participate in the allergic airway inflammation, the ovalbumin-induced pulmonary remodeling, and the RSV-induced AHR. Previous studies showed that PCF degeneration by neonatal capsaicin (CAP) pretreatment decreased the allergic airway inflammation [10] and the pulmonary remodeling characterized by collagen and elastic fiber deposition in airways [11] in ovalbumin-induced asthma model. PCF degeneration also eliminated the AHR induced by respiratory syncytial viral infection [12]. These PCF effects are thought to be achieved by the PCF-released neuropeptides, especially the substance P (SP) that is responsible for neurogenic inflammation, potentiation of airway constriction, epithelial cells proliferation, and mucus hypersecretion in asthma [13–16]. However, their roles in the pathogenesis of the HDM-induced asthma remain unexplored. While the HDM-induced asthma possesses the asthmatic characteristics (AHR, airway inflammation and remodeling) similar to those induced by OVA, ozone, and virus; however, the pathological processing to cause these characteristics is likely not the same. For example, ozone and respiratory syncytial virus either fails or only affects some types of inflammatory cells [12, 17], but HDM increases all or more types of inflammatory cells in our pilot and previously reported studies [9, 17, 18]. Moreover, HDM induces chronic airway inflammation and remodeling with a multifaceted immune responses initiated in the lungs, but the classical OVA model is more related to acute airway inflammation with the Th2 skewing adjuvant aluminum hydroxide and sensitization originated in the periphery [8]. Most importantly, HDM has shown to be able to uniquely and directly stimulate the cell bodies of PCFs resident in the nodose ganglion [19]. These lines of information encourage us to test the PCF involvement in in the HDM-induced AHR, airway remodeling, and airway inflammation. Revealing a causal impact of PCFs on airway remodeling including ASM, epithelial cells (Clara cells and ciliated cells), and mucus is novel as this PCF impact was not investigated in previous animal models. To examine our hypothesis, we evaluated the roles of PCFs in developing the chronic HDM exposure-induced AHR, airway remodeling (airway smooth muscle, airway epithelial cells, mucus secretion), and airway inflammation (BALF cells) in mice.

## Methods

### Ethics statement

All animals were managed in accordance with the Guide for the Care and Use of Laboratory Animals and approved by the Institutional Animal Care and Use Committee (IACUC), which is accredited by the Association for Assessment and Accreditation of Laboratory Animal Care International, USA (protocol# FY15-024).

### Animals

Adult female and male BALB/c mice were purchased from Charles River Laboratories, Inc. (Wilmington, MA), housed and mated in ABSL2 animal facility at Lovelace Respiratory Research Institute. Mouse pups born by spontaneous vaginal delivery were housed with their mother and siblings (24-25 °C, and 12:12 h light/dark cycle). In all experiments, no more than three female pups from each litter with similar overall litter size were used in each study series to minimize the possible effect of genetic difference between litters on the results.

### Animal pretreatments

#### Degeneration of c-fibers

Capsaicin (CAP, 50 mg/kg diluted in the solution containing 18 μl saline, 1 μl ethanol, and 1 μl Tween 80) or its vehicle was injected subcutaneously one or 2 days after birth [20]. The females were exposed to HDM or saline at 6-8 weeks old and correspondingly grouped as Ctrl_Intact_, HDM_Intact_, Ctrl_PCFX,_ and HDM_PCFX_. To ensure the viability of the C-fiber degeneration after CAP pretreatment, one drop (10 μl) of 0.01% capsaicin solution was applied onto the cornea at 6-8 weeks old. All vehicle pretreated mice displayed rigorous eye-wiping (> 30 wipes in 30 s), while those pretreated with CAP were virtually unresponsive or had fewer than 5 wipes with longer latencies.

#### Antigen administration

Female BALB/c mice pretreated with vehicle or CAP at 6-8 weeks old were exposed to HDM whole body extracts (Greer Laboratories, Lenoir, NC, USA. XPB82D3A25) intranasally (25 μg HDM protein diluted in 10 μl saline, HDM group) or saline (Ctrl group) every day for five consecutive days followed by 2 days rest and this treatment was repeated for five consecutive weeks [8]. Pups were randomly assigned to five *Study Series* as described below.

### Experimental protocols

*Study Series I* was designed to study the effects of PCF degeneration on the HDM-induced AHR. Ctrl_Intact_, HDM_Intact_, Ctrl_PCFX_, and HDM_PCFX_ mice (*n* = 12, 12, 9, and 9) were placed in the chamber. After stabilization, the sRaw responses to aerosolized saline and methacholine solutions were recorded.

*Study Series II* aimed to clarify the HDM effect on pulmonary SP and its dependency on PCFs. We detected the SP level in lung homogenates by ELISA in Ctrl_Intact_, HDM_Intact_, Ctrl_PCFX_, and HDM_PCFX_ mice (*n* = 7, 7, 5, 5). The upper lobe of the left lung was collected and homogenized; then supernatant was harvested.

*Series III* was conducted to study the effect of PCF degeneration on the HDM-induced airway smooth muscle remodeling. We compared the expression of airway smooth muscle by α-SMA immunofluorescence that was quantified by morphological analysis and western blot among the four groups of mice (*n* = 5/group, respectively). To functionally confirm the effect of PCFs on the contractile function of airway smooth muscle, we compared the tracheal contraction evoked by high potassium (67 mM) and methacholine in vitro among the four groups (*n* = 6/group).

*Study Series IV* was carried out to study the effect of PCF degeneration on the HDM-induced airway inflammation. Total cells and percentage of differential cell in BALF were counted (*n* = 7/group) and compared among the four groups of mice, while the morphology changes of airway and lung were detected by H&E stain (*n* = 3/group).

*Study Series V* was applied to study the influence of PCF degeneration on the HDM-induced changes in airway epithelial cells and airway mucus secretion. In four groups of mice, Clara cells (CCSP-positive) and ciliated cells (tubulin-positive) were labeled by immunofluorescence and quantified by morphological analysis. Moreover, airway mucus secretion was detected by AB-PAS satin (*n* = 6/group).

### Measurement of airway Hyperresponsiveness (AHR)

During the final 3 days of exposure, the mice were individually placed in a double-chamber whole body plethysmograph (Buxco Electronics, Inc., Wilmington, USA) for 30 min × 2 times daily for environmental conditioning. After the final exposure, AHR was measured in conscious mice by means of specific airway resistance (sRaw). In brief, the mice were individually placed in the double-chamber to assess sRaw [12]. The mice were exposed to aerosolized saline (0 mg/ml) and methacholine solutions in a dose-increasing manner (3.125, 6.25, 12.5, 25, and 50 mg/ml; Sigma). Each exposure was last for 1 min and apart from a 4 min-interval between the neighboring two exposures.

### Measurement of tracheal constriction in vitro

Tracheal segments were collected 48-72 h after the final HDM or saline exposure. Following removal of the surrounding tissues, the trachea (approximately 5 mm long) was cut below the larynx and above the bronchial bifurcation and gently mounted on two L-shaped metal prongs. The lower support was fixed and the upper support was connected to a force transducer (model FT03; Grass Instruments, Quincy, MA, USA) linked to a PowerLab data acquisition system (AD Instruments Ltd) via a preamplifier. The trachea was then immersed in a 10 ml tissue bath (model 47,264, World Precision Instruments) filled with normal physiological salt solution (PSS) (in mM: 119 NaCl, 4.7 KCl, 1.18 KH_2_PO4,1.17 MgSO4, 18 NaHCO3, 0.026 EDTA, 2.0 CaCl_2_, 11 Glucose and 12.5 Sucrose) that was maintained at 37 °C and constantly bubbled with 95%/5% of O_2_/CO_2_ mixture. The resting tension of 0.5 g was applied and maintained during a 60 min equilibration period with changes of fresh PSS solution every 15-20 min. Following equilibration, the trachea was challenged with a high potassium PSS solution (67 mM KCl, in mM: 56.7 NaCl and 67 KCl, others same as above PSS). When the contraction reached a steady state for 5-10 min, the tissue bath was rinsed with normal PSS to completely relax the trachea. After another 20 min equilibration at 0.5 g resting tension, the trachea was challenged with cumulative concentrations of methacholine (10^−9^-10^−4^ M). Concentration of methacholine solution was increased every 10-15 min or until the contraction approached a plateau [21]. Data are normalized to baseline level.

### Bronchoalveolar lavage fluid (BALF) and cell count

The mice were euthanized with 1:9 diluted Euthasol (150-200 mg/kg, intraperitoneally) 48-72 h after the final HDM or saline exposure. The trachea was cannulated with a polyethylene tube (Becton Dickinson, Sparks, MD). Prior to lavage, the left bronchus was tied and the right lung was lavaged three times with 0.5 ml cold saline for collecting BALF. Approximately 1.3 ml of the instilled fluid was consistently recovered. The BALF samples were centrifuged for 5 min at 1500 rpm at 4 °C. Supernatants were decanted and immediately frozen at −80°. Cell pellets were resuspended in saline. The total cell numbers were counted using a hematocytometer. BALF cell smears (100 μL) were prepared using cytospin apparatus and stained with Diff-Quik solution (Life Technologies, Auckland, New Zealand) to determine the differential cells counts in accordance with conventional morphological criteria. At least 300 cells per slide were evaluated in order to obtain the differential cell counts.

### Histology and Immunofluorescence

The left lobes were inflated with 4% paraformaldehyde at 25 cm H_2_O pressure. Paraffin-embedded tissue sections (5 μm) were then prepared and the sections were evenly divided into three groups. Sections were stained with hematoxylin and eosin (H&E) or stained with Alcian blue and PAS (AB-PAS).

For immunofluorescence staining, paraffin-embedded sections were dewaxed through xylene (2 changes) and rehydrated through descending alcohol (100%-95%-80%-70%) to deionized H_2_O. Antigen recovery was performed by using pre-heated Na-Citrate buffer (10 mM, pH 6.0) for 10 min in the microwave. Sections were blocked in blocking buffer (3% BSA, 0.3% Triton X-100 in PBS) at room temperature for 1 h. Sections were incubated with primary antibody at 4 °C overnight, incubated with secondary antibody at room temperature for 1 h. Cover slips were mounted on stained sections with anti-fade reagent containing DAPI (Invitrogen, USA). The primary antibody used was Anti-Clara cell secretory protein (CCSP also known as CC10 OR CC16) (1:2000 dilution, 07-623, EMD Millipore), monoclonal anti-acetylated tubulin (1:10,000 dilution, T7451, Sigma), and monoclonal anti-α smooth muscle actin (1:400 dilution, A 2547, Sigma). The secondary antibody used was Alexa Fluor labeled goat anti-rabbit antibody, goat anti-mouse antibody (1:200 dilution, A-11008, A-11005, Thermo Fisher, USA).

### Morphometry analysis

Digital images of the immunohistochemistry were obtained with the use of a light microscope (ZEISS) equipped with a digital camera (ZEISS) linked to a computer, and then analyzed with the use of ImageJ software (NIH, Bethesda, MD, USA). This was used because histologic analysis reduces three-dimensional structures to two dimensions in which volumes become areas and surfaces become lines. The ratio of tissue area to the length of the basement membrane was used to express the ratio of the volume to the surface area (V:SA) or the thickness of the airway wall and its compartments [5, 22].

Airway smooth muscle remodeling was evaluated by measuring the thickness of airway smooth muscle, defined by the ratio of the α-SMA positive area to the length of the subepithelial basement membrane. The expression of epithelial cells (Clara cells and ciliated cells) was evaluated by measuring the ratio of the volume to the surface area (V:SA), which is defined by the ratio of the epithelial cell positive area to the length of the subepithelial basement membrane. Five [7] airways per mouse were quantified and averaged.

### ELISA

The SP levels in lung homogenates were measured by commercial ELISA kit (Cayman). The protocols were followed according to the manufacturer’s instructions. The upper lobe of the left lung was collected, ground and homogenized with M2 lysis buffer (v:v 1:9) containing protease inhibitor PMSF, followed by centrifugation at 13000 rpm for 5 min at 4 °C. The supernatant was harvested and stored at −80 °C.

### Western blot

The trachea and bronchi tissues were homogenized with M2 buffer (20 mM Tris-HCl pH 7.6, 0.5% NP40, 250 mM NaCl, 3 mM EDTA, 2 mM DTT, 0.5 mM phenylmethylsulfonylfluoride, 20 mM β-glycerophosphate, 1 mM sodium vanadate, and 1 μg/ml leupeptin). Protein concentrations were detected by using the BCA assay reagent (Bioteke). Equal quantities of protein homogenates were run in a 12% SDS-PAGE (sodium dodecyl sulfate polyacrylamide gel electrophoresis) and then transferred to PVDF membranes (Millipore, Billerica, MA). The membranes were probed with primary antibody overnight at 4 °C and followed by a second antibody for 1 h at room temperature. Signals were detected by enhanced chemiluminescence according to the manuals (Millipore, Billerica, MA). The band density was quantified using ImageJ software (NIH, Bethesda, MD, USA) and normalized relative to GAPDH. The primary antibody used was monoclonal anti-α smooth muscle actin (1:2000 dilution, mouse, A 2547, Sigma), GAPDH (1:2000 dilution, mouse; sc-32,233, Santa Cruz, CA, USA). The secondary antibody used was alkaline phosphatase-conjugated goat anti-mouse antibody (1:2000 dilution, Santa Cruz, CA, USA).

### Data analysis

Data were analyzed using GraphPad Prism 6.0 software (GraphPad, San Diego, CA) and presented as mean ± SEM. Statistical significance was assessed by two-way analysis of variance (ANOVA) to assess significant differences of the tested variables among the four groups. Tukey’s test was utilized for specific comparisons between individual groups. Any *P* values < 0.05 were considered statistically significant.

## Results

### PCF degeneration decreased the HDM-induced AHR.

**Fig. 1:**
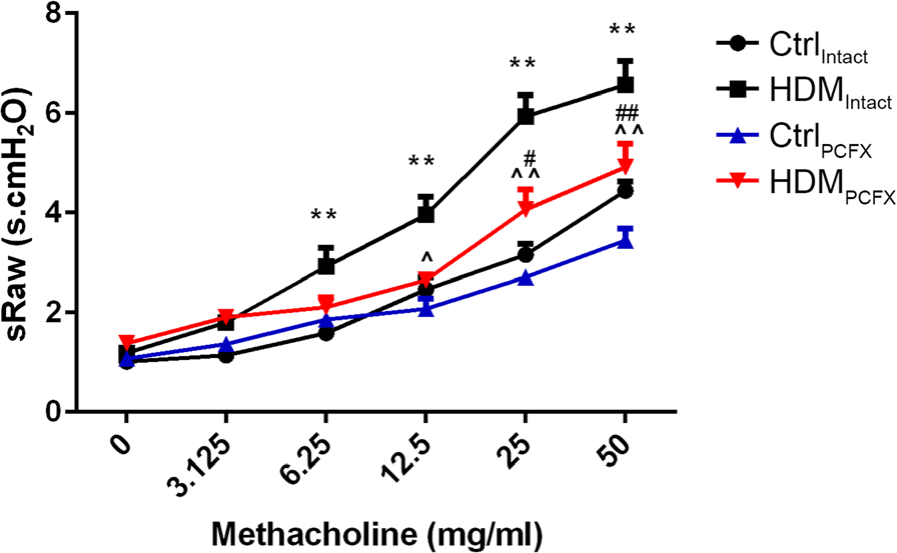
Effects of degeneration of pulmonary C-fibers (PCFs) on the house dust mite (HDM)-induced airway hyperresponsiveness (AHR). Airway resistance (sRaw) was measured during exposure to aerosolized saline (0 mg/ml) and methacholine solutions in a dose-increasing manner. Numbers of mice were 12, 12, 9, and 9 for Ctrl_Intact_, HDM_Intact_, Ctrl_PCFX_, and HDM_PCFX_ respectively. Data are presented as mean ± SEM. ** *P* < 0.01, HDM_Intact_ versus Ctrl_Intact_ group. # and ## *P* < 0.05 and 0.01, HDM_PCFX_ versus Ctrl_PCFX_ group. ^ and ^^, *P* < 0.05 and 0.01, HDM_PCFX_ versus HDM_Intact_ group

To investigate whether PCF degeneration affects the HDM-induced AHR, we compared the responses of airway resistance to increasing doses of methacholine challenge among Ctrl_Intact_, HDM_Intact_, Ctrl_PCFX_, and HDM_PCFX_ mice. sRaw responses to methacholine concentrations at 6.25, 12.5, 25, and 50 mg/ml were significantly higher in HDM than Ctrl mice in the intact group (Fig. 1). Interestingly, this HDM-evoked AHR was almost eliminated by PCF degeneration. In contrast, PCF degeneration did not markedly alter the sRaw response in Ctrl mice. It is worthy to note that no significant difference of body weights (19.7 ± 0.9 g vs. 19.8 ± 0.7 g) and baseline ventilation (65.3 ± 6.4 ml/min vs. 63.6 ± 8.8 ml/min) was observed between Ctrl_PCFX_ and Ctrl_Intact_ groups, pointing to the lack of PCF degeneration impact on normal physiological function.

### PCF degeneration reduced pulmonary substance P levels.

**Fig. 2:**
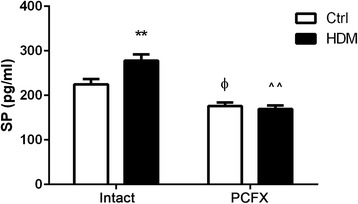
Substance P (SP) levels in lung homogenates. SP levels were measured by ELISA. Data are presented as mean ± SEM. Numbers of mice were 7, 7, 5, and 5 for Ctrl_Intact_, HDM_Intact_, Ctrl_PCFX_, and HDM_PCFX_ respectively; ** *P* < 0.01, HDM_Intact_ versus Ctrl_Intact_ group; ^ϕ^ *P* < 0.05, Ctrl_PCFX_ versus Ctrl_Intact_ group; ^^ *p* < 0.01, HDM_PCFX_ versus HDM_Intact_ group

To understand HDM effect on the PCF-originated pulmonary SP, we tested the SP levels in the lungs using ELISA in the four groups of mice. As shown in Fig. 2, HDM profoundly elevated pulmonary SP in the intact mice, while this HDM-evoked response disappeared after PCF degeneration (no significant difference of SP between HDM_PCFX_ and Ctrl_PCFX_ mice). Furthermore, the SP level in Ctrl mice was significantly lowered after PCF degeneration. These data suggest a PCF-origination of the pulmonary SP, especially SP response to HDM.

### PCF degeneration attenuated the HDM-induced airway smooth muscle remodeling.

**Fig. 3:**
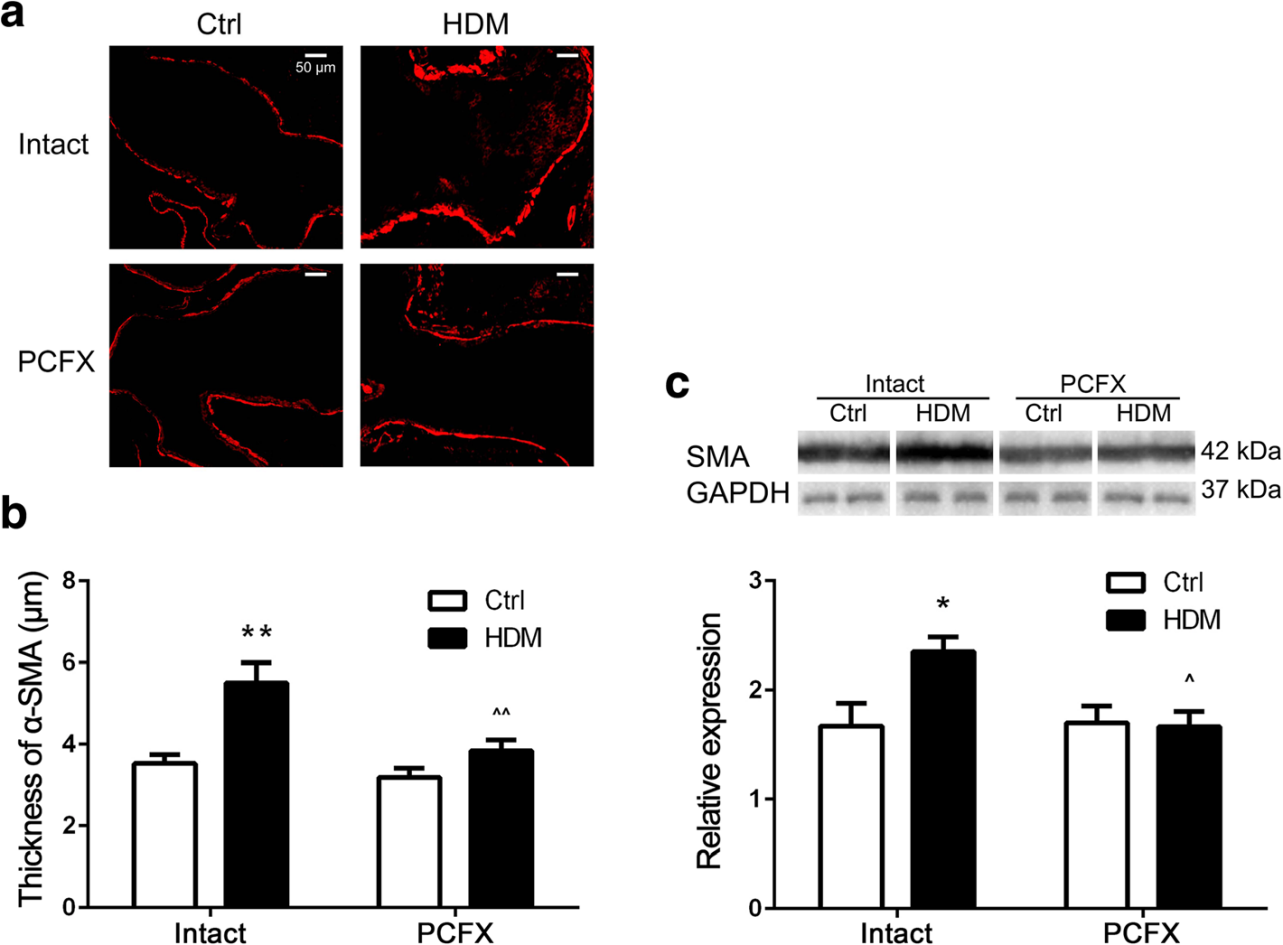
Effects of degeneration of pulmonary C-fibers (PCFs) on the house dust mite (HDM)-induced airway smooth muscle (ASM) proliferation. **a** Immunofluorescence for α-SMA (red) in the airways (scale bars = 50 μm). Images are the micrographs showing the representative α-SMA immunoreactivity in a Ctrl_Intact_, HDM_Intact_, Ctrl_PCFX_, and HDM_PCFX_ mice, respectively. **b** Quantification of α-SMA in the airways. The thickness of airway smooth muscle layer was expressed as the ratio of α-SMA positive area to the length of the subepithelial basement membrane. Data are presented as mean ± SEM. Numbers of mice were five in each group. **c** α-SMA quantified by western blot and normalized to GAPDH levels. Numbers of mice were five in each group; * and ** *P* < 0.05 and 0.01, HDM_Intact_ versus Ctrl_Intact_ group; ^ and ^^ *P* < 0.05 and 0.01, HDM_PCFX_ versus HDM_Intact_ group

Airway smooth muscle plays an important role in AHR of asthma. In order to investigate the effect of PCF degeneration on expression of airway smooth muscle, we used α-SMA to label airway smooth muscle by immunofluorescence. We found the highest density and continuousness of airway α-SMA expression in HDM_Intact_ mice among the four groups (Fig. 3a). In other words, α-SMA expression in the airway was increased by HDM and the response was diminished by PCF degeneration. Statistically, the thickness of airway smooth muscle was significantly higher in HDM_Intact_ than Ctrl_Intact_ mice and the thickened airway smooth muscle by HDM disappeared after PCF degeneration (i.e., no difference between Ctrl_Intact_ and HDM_PCFX;_ Fig. 3b). The similar results were also observed in expression of airway smooth muscle quantified by western blot (Fig. 3c).

### PCF degeneration failed to affect tracheal contraction evoked by high potassium and methacholine in vitro.

**Fig. 4:**
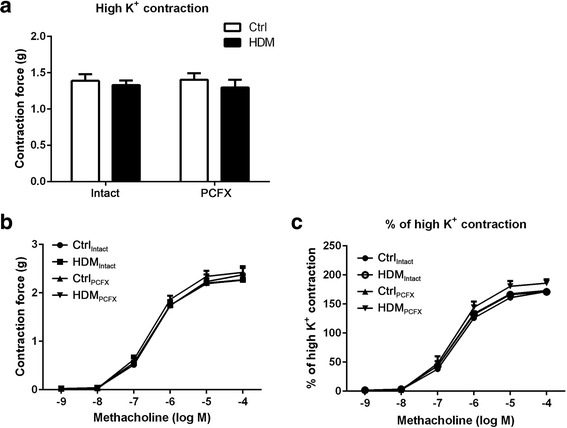
Tracheal contraction evoked by high potassium (67 mM) and methacholine in vitro. **a** High potassium (67 mM) contractile response of tracheal ring. **b** Tracheal ring contractile response curves to 10^−9^-10^−4^ M methacholine. **c** The methacholine-induced contractile response normalized to the high potassium (67 mM) response. Data are presented as mean ± SEM. Numbers of mice were six per group

In this study, we asked if HDM-exposure would increase the contractility of airway smooth muscle in response to high potassium (67 mM) and methacholine in vitro and what the role of PCFs was in the contractility. Unexpectedly, we observed that the tracheal contraction evoked by high potassium (67 mM) was similar in the four groups (Fig. 4a). Moreover, the contraction of the tracheal ring was gradually increased during exposure to methacholine concentrations from 10^−9^ M to 10^−4^ M as exhibited in Fig. 4b and c. The contraction increased obviously at methacholine 10^−7^ M, and approached plateau at 10^−5^ M, but there was no significant difference between the responses among the four groups.

### PCF degeneration altered the HDM-induced airway inflammation.

**Fig. 5:**
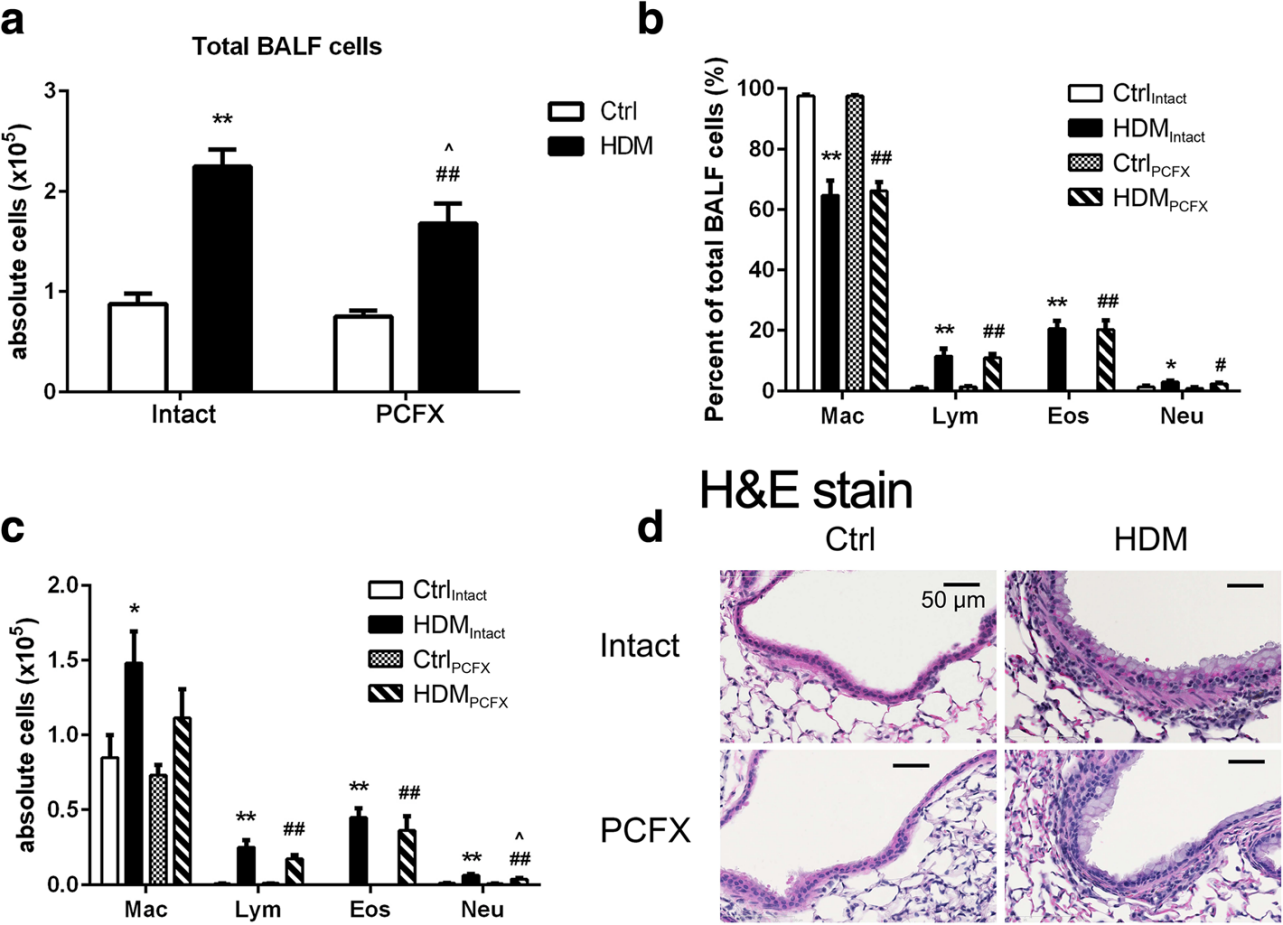
The effect of degeneration of pulmonary C-fibers (PCFs) on the house dust mite (HDM)-induced inflammatory response in the lungs. **a** Total BALF cells count. **b** Percentage of macrophages, lymphocytes, eosinophils and neutrophils in BALF. **c** Numbers of cells of each cell type. **d** H&E stain. Images are representative H&E strained lung sections from each group (bars = 50 μm). Results are expressed as mean ± SEM. Numbers of mice were seven in each group; ** *P* < 0.01, HDM_Intact_ versus Ctrl_Intact_ group. ## *P* < 0.01, HDM_PCFX_ versus Ctrl_PCFX_ group. ^ *P* < 0.05 HDM_PCFX_ versus HDM_Intact_ group. Mac = macrophages; Lym = lymphocytes; Eos = eosinophils; Neu = neutrophils

Airway chronic inflammation is an evident character of asthma. Figure 5a showed that HDM exposure significantly doubled total BALF cells and this response was markedly reduced by PCF degeneration. In contrast, PCF degeneration did not change the total BALF cell count in Ctrl mice. With respect to the cell differentiations, HDM exposure strikingly elevated the percentages of lymphocytes, eosinophils and neutrophils in total BALF cells, especially eosinophils, and these changes were unaffected by PCF degeneration (Fig. 5b). We also examined the absolute cell number in each cell type and found that PCF degeneration significantly reduced the absolute cell number of neutrophils in BALF with little changes in other cell types (Fig. 5c). Morphologically, cell infiltration in the airways and lungs induced by HDM exposure were not profoundly altered by PCF degeneration (Fig. 5d).

### PCF degeneration did not significantly affect the HDM-induced changes in epithelial cells and mucus secretion.

**Fig. 6:**
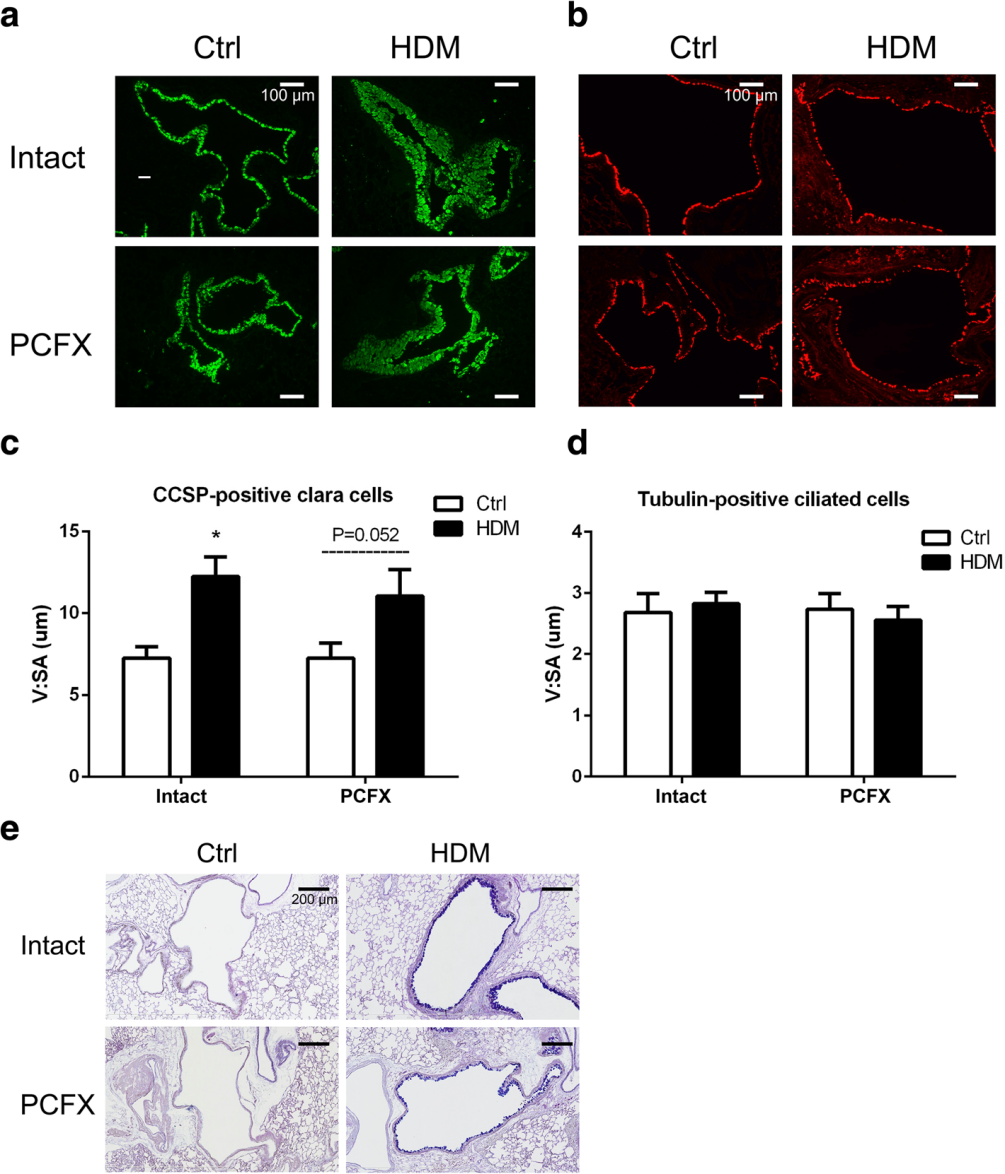
The immunofluorescent expression of epithelial cells (Clara cells, ciliated cells) and airway mucus. Examples of CCSP-positive Clara cells (green) and their group data are illustrated in (**a**) and (**c**) with the bar equal to 100 μm, while examples of Tubulin-positive ciliated cells (red) and their group data in (**b**) and (**d**). V:SA is the ratio of the volume to the surface area. In C, results are expressed as mean ± SEM. Numbers of mice were six in each group; * *P* < 0.05, HDM_Intact_ versus Ctrl_Intact_ group. And *P* = 0.052, HDM_PCFX_ versus Ctrl_PCFX_ group. **e** AB-PAS stain (bars = 200 μm). Images are representative micrographs of lung tissues obtained from each group

To quantify the change of epithelial cells induced by HDM and the role of PCFs in these changes, CCSP-labeled Clara cells and tubulin-labeled ciliated cells in the airways were stained by immunofluorescence (Fig. 6a and b). As presented in Fig. 6a and c, the layer of CCSP-positive Clara cells was significantly thickened and highly hypertrophied with stratified-like aspect by HDM exposure with limited effect produced by PCF degeneration. Different from CCSP-labeled Clara cells, neither HDM exposure nor PCF degeneration affected the expression of tubulin-positive ciliated cells (Fig. 6b and d). Parallel to the data of CCSP-positive Clara cells, HDM exposure induced a remarkable increase in mucus production independent of PCFs (Fig. 6e).

## Discussion

PCF degeneration has shown an abolishment of airway resistance response to allergic stimulations by ovalbumin [23] and ozone [17]. Our finding that PCF degeneration eliminated the HDM-induced AHR convincingly demonstrates a critical involvement of PCFs in the AHR response to HDM exposure, which is novel. To probe the mechanisms underlying the PCFs’ contribution to the HDM-induced AHR, we detected the level of pulmonary SP. The latter is primarily synthesized in the cell bodies of PCFs [11] and released locally from peripheral sensory terminals upon stimulation [24]. It is responsible for airway/lung neurogenic inflammation, promotes ASM contraction, AHR, mucus hypersecretion and inflammation in asthma [13, 14, 16]. In fact, blockade of vesicle release/recycling machinery of lung sensory neurons to minimize neuropeptides’ release (including SP) inhibits the allergic AHR induced by ovalbumin [25]. In the present study, we found a PCF-dependent increase of pulmonary SP in HDM-exposed mice, supporting a stimulatory impact of HDM in the release of SP from PCFs. It has been unclear how PCFs participate in the AHR to HDM-exposure; however, two factors are likely involved. First, the cell bodies of PCFs residing in the nodose ganglion could be stimulated by HDM acting via PAR_2_ receptors in mice [19]. Interestingly, PAR_2_ is expressed in PCFs and its activation synergizes the transient receptor potential vanilloid 1 (TRPV1)-mediated responses [26]. Second, vagal TRPV1 may be responsible for the HDM-induced AHR because selective ablation of TRPV1-neurons in the vagal ganglion completely prevented AHR induced by ovalbumin [25]. Recent reports have revealed an interaction of SP receptor (neurokinin 1 receptor, NK1R) and TRPV1. The majority of TRPV1 positive neurons co-express NK1R, and NK1R can potentiate TRPV1 activity [27, 28]. Therefore, we reason that the critical role PCFs play in the HDM-induced AHR is, at least in part, realized by the direct HDM stimulation of PCFs via acting on PAR_2_ and PCFs’ released SP acting on NK1R to synergize the TRPV1.

We next tested the role of PCF degeneration on ASM change induced by HDM. Similar to a previous report in mice [8], we found that HDM increased AMS thickening and more importantly that PCF degeneration almost eliminated the HDM-induced ASM thickening, indicating PCF involvement in the mechanism of ASM remodeling. The assumption of the PCF involvement in the development of ASM thickening by releasing SP is supported by following findings. First, SP can induce ASM cell proliferation in vitro [29, 30]. Second, SP induces ASM contraction [14] to produce mechanical force on airway structures that may, in turn, evoke airway remodeling, such as elevated ASM thickening [31]. To reveal the effect of HDM on the contractile function of ASM, we examined tracheal contraction in vitro. Unexpectedly, HDM exposure did not increase tracheal contraction response to cholinergic agonist (methacholine). This finding is consistent with a report showing no difference between tracheal response to methacholine in ovalbumin immunized and nonimmune mice, although acetylcholine (ACh) release was significantly increased in the former [32]. In fact, the dominant role of PCFs in the HDM-induced AHR as noted in this study may account for the lack of difference in tracheal contraction response to methacholine in vitro. In other words, PCF tonic over-excitation required for the HDM-induced AHR is absent in vitro. Additionally, bronchial constriction is known to be the major contributor to the AHR [33], but its contribution was not examined in vitro in the present study. Recent studies reveal a crucial role of the abnormality of Abelson tyrosine kinase [34] and glycogen synthase kinase 3 beta [35, 36] in generating ASM hyperplasia (airway remodeling) and airway inflammation in asthmatic mice models. Further investigation is needed to verify whether this abnormality is involved in HDM-induced airway inflammation and ASM hyperplasia and whether PCF degeneration is able to prevent or diminish this abnormality.

Airway remodeling also includes thickened abnormal airway epithelium with mucus gland hypertrophy that is characterized by hypersecretion in goblet cells in patients with asthma. In mice, Clara cells are the predominant secretory cell type of airway epithelial cells [37]. It is known that ovalbumin-exposure can induce mucous metaplasia of airways in Clara cells and the latter produces mucin in mice [38, 39]. However, it is unknown whether and how HDM-exposure affects Clara cells although HDM-induced mucus hypersecretion has been identified in mice. Thus, we inspected the morphological changes of epithelial cells (Clara cells and ciliated cells) and mucus secretion in HDM-exposed airways. Interestingly, HDM exposure induced Clara cell layer thickening and mucus hypersecretion without a significant change in the thickness of ciliated cells layer. The pathophysiological functions of these changes evoked by HDM exposure are unclear but may be related to strengthening airway epithelium defensive barrier. Previous studies have shown anti-inflammatory functions of CCSP in asthma, instead of labeling Clara cells [39], and the protective influence of Clara cells on airway epithelium via secreting surfactant proteins and anti-proteinases [40], along with trapping inhaled pathogens by mucus. Interestingly, we found that PCF degeneration did not reverse the changed Clara cells and mucus hypersecretion induced by HDM, suggesting that airway epithelium may not be the main target of PCFs in the HDM-induced asthma model.

Airway inflammation is a prominent feature of asthma, involving multiple inflammatory cells and cellular mediators. These inflammatory cells in asthmatic airways include eosinophils, T lymphocytes, macrophages, and neutrophils [4]. We found that HDM exposure increased inflammatory cells including elevation of total BALF cells and absolute numbers of eosinophils, lymphocytes, neutrophils and macrophages in BALF, which is similar to a previous report [9, 18]. The new finding here is that PCF degeneration significantly reduced the total BALF cells and the number of neutrophils. Our finding demonstrates a modulatory effect of PCFs on airway inflammatory cell response to HDM, confirming a neuro-immune interaction in asthmatic development. Neuropeptides are suggested as the key mediators of a neuro-immune pathway [41]. It is well documented that SP mainly released from PCFs mediates the neurogenic inflammation in the airways [14] and contributes to the neuro-immune crosstalk in asthma [16]. Therefore, the PCF degeneration-induced changes in inflammatory cell responses to HDM exposure may be related to SP. We conclude that PCFs modulate neurogenic inflammation by releasing neuropeptides, such as SP, and participate in the mechanism of allergic airway inflammation induced by HDM.

## Conclusion

In summary, our major findings in this study are that PCFs degeneration: 1) decreases the HDM-induced AHR with the pulmonary SP response to HDM diminished; 2) reduces HDM-induced ASM mass in airway remodeling; 3) lowers BALF cells, especially neutrophils, in response to HDM without effect on the percentage of different cells; and 4) fails to significantly affect the epithelium thickening and mucus hypersecretion. Our results have translational significance. In the clinical setting, inhaled corticosteroids are currently the most effective anti-inflammatory medication; however, it has limited effect on protecting against airway remodeling [31, 42]. Our data show the key role PCFs play in the genesis of allergic asthma, which highlights a new potential way to therapeutically intervene ASM remodeling by inhibition of PCFs.

## Abbreviations

AHR: Airway hyperresponsiveness
ASM: Airway smooth muscle
BALF: Bronchoalveolar lavage fluid
CAP: Capsaicin
CCSP: Clara cell secretory protein
GAPDH: Glyceraldehyde-3-phosphate dehydrogenase
HDM: House dust mite
NK1R: Neurokinin receptor 1
PAR_2_: Protease-activated receptor 2
PCFs: Bronchopulmonary C-fibers
PSS: Physiological salt solution
SP: Substance P
sRaw: Specific airway resistance
TRPV1: Transient receptor potential vanilloid 1
